# Cerebellar Asymmetry and Cortical Connectivity in Monozygotic Twins with Discordant Handedness

**DOI:** 10.1007/s12311-017-0889-y

**Published:** 2017-10-23

**Authors:** R. E. Rosch, P. E. Cowell, J. M. Gurd

**Affiliations:** 1 Wellcome Trust Centre for Neuroimaging, Institute of Neurology, University College London, London, UK; 2 Developmental Neurosciences Programme, UCL Great Ormond Street Institute of Child Health, University College London, London, UK; 3 Department of Human Communication Sciences, University of Sheffield, 362 Mushroom Lane, Sheffield, S10 2TS UK; 4 Nuffield Department of Clinical Neurosciences, University of Oxford, Oxford, UK

**Keywords:** Cerebellum, Corpus callosum, Twins, monozygotic, Functional laterality, Neuroimaging

## Abstract

Handedness differentiates patterns of neural asymmetry and interhemispheric connectivity in cortical systems that underpin manual and language functions. Contemporary models of cerebellar function incorporate complex motor behaviour and higher-order cognition, expanding upon earlier, traditional associations between the cerebellum and motor control. Structural MRI defined cerebellar volume asymmetries and correlations with corpus callosum (CC) size were compared in 19 pairs of adult female monozygotic twins strongly discordant for handedness (MZHd). Volume and asymmetry of cerebellar lobules were obtained using automated parcellation.CC area and regional widths were obtained from midsagittal planimetric measurements. Within the cerebellum and CC, neurofunctional distinctions were drawn between motor and higher-order cognitive systems. Relationships amongst regional cerebellar asymmetry and cortical connectivity (as indicated by CC widths) were investigated. Interactions between hemisphere and handedness in the anterior cerebellum were due to a larger right-greater-than-left hemispheric asymmetry in right-handed (RH) compared to left-handed (LH) twins. In LH twins only, anterior cerebellar lobule volumes (IV, V) for motor control were associated with CC size, particularly in callosal regions associated with motor cortex connectivity. Superior posterior cerebellar lobule volumes (VI, Crus I, Crus II, VIIb) showed no correlation with CC size in either handedness group. These novel results reflected distinct patterns of cerebellar-cortical relationships delineated by specific CC regions and an anterior-posterior cerebellar topographical mapping. Hence, anterior cerebellar asymmetry may contribute to the greater degree of bilateral cortical organisation of frontal motor function in LH individuals.

## Introduction

Research on regional anatomy of the human cerebellum has revealed topographically defined functional distinctions and asymmetries [1]. Traditionally considered a component of the motor system [2, 3], emerging evidence links functions of individual cerebellar lobules to higher-order cognitive functions [4], such as language [5, 6], visuospatial attention [7], working memory [8–12] and performance monitoring [13, 14]. Moreover, cognitive [13–15] and cognitive-affective disturbances [16, 17] associated with cerebellar damage are more common than once assumed.

Anatomically, the dominant primary and horizontal fissures separate anterior, superior-posterior and inferior-posterior cerebellar lobes [18]. Subdivision of the human cerebellum into anterior (lobules I–V), superior-posterior (lobules VI, Crus I, Crus II, VIIb) and inferior-posterior cerebellar (lobules VIII, IX) lobes has more recently been used in fMRI mapping of motor function in healthy volunteers [19] and electrophysiological mapping of evoked motor responses in neurosurgical patients [20]. Anterior lobules are associated with ipsilateral control of simple, repetitive movements, but there is functional heterogeneity within the superior-posterior cerebellum. Higher-order cognition is associated with Crus I and Crus II, and a combination of complex motor and cognitive functions is linked to lobules VI and VIIb [1, 19–21].

The cerebellum derives much of its functional specificity from connectivity with cerebral cortex. Regarding lateralised function, cerebellar asymmetry broadly corresponds to that of the contralateral cortical hemisphere [1], with relationships between cortico-cerebellar circuits and functional asymmetry of the cerebellum that are increasingly documented [6, 22–25]. In the mammalian brain, right and left cerebellum are linked through common inputs from pontine nuclei [26] and their axonal projections [27]. However, there are no direct, intrinsic connections between the two cerebellar hemispheres [28]. Therefore, cortical structures such as the corpus callosum (CC) which mediate cerebral asymmetry may be salient to the organisation of cerebellar asymmetry and to lateralised cortico-cerebellar functional networks.

CC fibre tracts have been mapped to outlying cortical areas with particular structural and functional significance [29–31]. Neuroimaging studies highlight the CC’s role in the organisation of lateralised cortical systems [32, 33], which have revealed different patterns of neurobehavioural correlations in right and left handers [34–36]. Similarly, in monozygotic handedness discordant (MZHd) twins, Gurd et al. [37] revealed larger anterior CC size to be associated with atypical lateralisation in frontal lobe activation during covert verbal fluency, a pattern more frequently found in left handers. CC mediated differences between right- and left-handed singletons have also been described in the ipsilateral deactivation of motor cortex during finger tapping, with more bilateral deactivation in left handers [38].

Human brain asymmetries are shaped by combined genetic, environmental and epigenetic effects over time, as revealed by studies of twins [39, 40]. MZ twins discordant for particular features afford further insights into genetic and environmental interactions and their impact on neurocognitive organisation [41] and mental health [42]. Left handers show a higher prevalence of atypical cortical functional lateralisation than right handers [43–45]. In the current study, these research strands on twins, handedness and brain asymmetry are extended to the cerebellum. At the interface of motor behaviour and cognition, the cerebellum is an excellent candidate for the study of adaptive motor function. Its links with cortex may hold clues to the neurodevelopmental basis of handedness, and MZHd twins provide a unique paradigm for probing these phenomena.

## Methods

### Participants

The participants were 19 pairs of female adult MZHd twins selected from full data sets previously described [37, 46]. Informed consent was obtained from all individual participants included in the study. Mean age was 52.37 years (SD 8.87, range 37–67). Right-handed (RH) and left-handed (LH) discordance was based on strong hand preference for writing. Mean Edinburgh Hand Preference Inventory (HPI) (range −100 to +100) for RH twins was 95.79 (SD 9.61, median 100, range +70 to +100) and for LH twins was −70.89 (SD 43.76, median −90, range +20 to −100). Twins were matched for IQ (RH mean 116.63, SD 11.94; LH mean 117.68, SD 11.27; paired *t* test, *t*(18) = −0.74, n.s.). This study was approved by the Central Oxfordshire Regional Ethics Committee (COREC).

### Image Acquisition

The structural magnetic resonance images were acquired on a 1.5-T Magnetom SONATA (Siemens, Erlangen, Germany) MRI scanner. The anatomical whole-brain images were obtained using a T1-weighted, 3D gradient echo-pulse sequence (FLASH, fast low-angle shot) with the following parameters: repetition time, 1200 ms; echo time, 5.6 ms; inversion time, 19° flip angle; matrix size, 160 × 256 × 208; voxel size, 1 mm isotropic; acquisition, coronal; and averages, 3.

### Image Analysis

Cerebellar volume analysis was conducted using the SUIT toolbox for cerebellum and brain stem [47] within SPM8 (www.fil.ion.ucl.ac.uk/spm/software/spm8/) on MathWorks MATLAB R2012a (www.mathworks.co.uk). Images were manually aligned so that anterior and posterior commissures were on the same axial plane. The SUIT toolbox was then used to automatically create brain stem and cerebellum isolation maps that were manually corrected where necessary. Utilising the manually corrected masks, every subject’s scan was normalised individually into SUIT space, resulting in a deformation map from individual anatomical space into SUIT space.

**Fig. 1:**
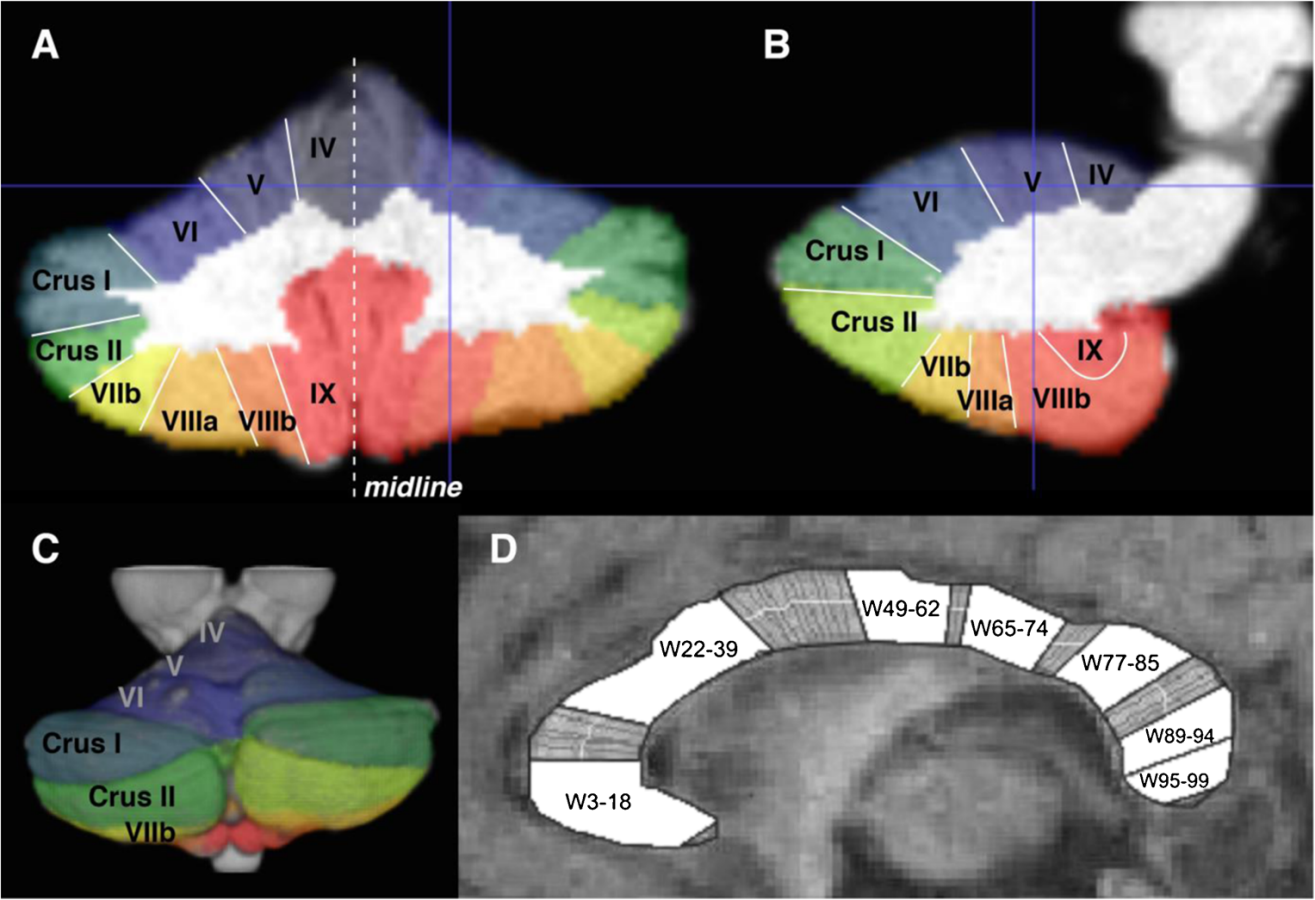
Parcellation of cerebellum and corpus callosum. **a**–**c** Automatic segmentation results using the SUIT toolbox in SPM8. Different colours mask individual lobule volumes in anatomical MRI space in **a** coronal, **b** sagittal and **c** 3D-reconstructed views. Crosshairs show right lobule V. **d** Midsagittal view of the corpus callosum (anterior = left of image) with regional clusters depicted in relation to the 99 percentile widths

These deformation maps were then used to reslice a probabilistic lobular atlas of the cerebellum [48] from SUIT space into each subject’s respective anatomical space (Fig. 1a–c). These individualised atlas images were then used to estimate the number of grey matter voxels for each of the lobules (anterior lobules IV and V; superior posterior lobules VI, Crus I, Crus II and VIIb; inferior posterior lobules VIIIa, VIIIb, IX and X) automatically using the SUIT toolbox. Estimates of hemispheric, not vermis, volumes were included in this analysis.

Lobular volumes are reported in mm^3^ (voxels of 1 × 1 × 1 mm each). Laterality indices were calculated as the difference between hemispheres normalised to total bihemispheric lobular volume (Eq. 1). This yields a measure of relative volume difference, with positive values indicating that left volumes are larger than right volumes:

1 $$ \mathrm{LI}=\frac{\mathrm{L}-\mathrm{R}}{\mathrm{L}+\mathrm{R}\ } $$

where LI is the laterality index, L is the left lobule volume, and R is the right lobule volume.

In addition to individual lobes, regions of interest were cerebellar areas involved in voluntary motor control (anterior regions) compared to those putatively involved in higher cognitive processing (superior-posterior regions). A distinction is often drawn between lobules V (anterior) and VI (posterior) with the divide formed by the primary fissure. This division accords with electrophysiological studies [20] and accounts for the involvement of lobule VI in cognitive and complex motor tasks in human imaging studies [1] and its psychophysiologically defined connections with prefrontal cortex [49]. Hence, the anterior (IV, V) and superior-posterior (VI, Crus I, Crus II, VIIb) lobules were analysed here.

CC midsagittal area, length and regional width measures were derived by manual tracing and subsequent automated parcellation using Callosum software (cf. [37]). The midsagittal CC was manually traced by a single rater (PEC) blind to the identity of participants. The software automatically divides the traced outlines into dorsal and ventral perimeters, and subsequently into percentiles along its anterior-posterior axis. Each CC was traced five times to average out variations due to manual-visual coordination and other forms of rater-based error. Coefficients of variation (CV = SD/mean) for the five tracings were computed for area, length and each of the 99 widths to confirm that values were lower than 10%. When CVs were greater than 10%, a new set of tracings was made. Mean values across the final five tracings were used in statistical analysis. In addition, the 99 dorsal-ventral width measurements were clustered into seven regions based on previously documented factor analysis (Fig. 1d) [50]: W3–18, W22–39, W49–62, W65–74, W77–85, W89–94 and W95–99. This factor structure has been replicated [51, 52].

### Statistical Data Analysis

Statistical analyses addressed the questions:

1. Does handedness affect volumetric asymmetry patterns observed in (a) anterior cerebellar lobules associated with cortical motor regions or (b) superior-posterior cerebellar lobules associated with prefrontal cortical regions?
2. Are there relationships between cerebellar asymmetries and the CC, and do they differ between LH and RH twins for (a) anterior cerebellar lobules and motor regions of the CC or (b) superior-posterior cerebellar volume asymmetries and prefrontal regions of the CC?
  Statistical analysis was conducted in SPSS (IBM version 21). To address questions 1a and 1b, volumetric measures for RH and LH twins were compared using repeated measures ANOVA. To analyse the relationships between regional cerebellar volume asymmetry and cortex (questions 2a and 2b), repeated measures ANCOVAs with regional cerebellar hemispheric volumes as the dependent measures and regional CC widths as covariates of interest were conducted.

## Results

### Cerebellar Volumes and Asymmetries

Mean individual lobular volumes and standard errors are shown in Table 1.

**Table 1:** Means and standard errors for cerebellar lobule volumes as a function of regional groupings, handedness and hemisphere

		Right-handed twins				Left-handed twins			
Region	Lobule	Left hemisphere	Right hemisphere	LI	*r*	Left hemisphere	Right hemisphere	LI	*r*
Anterior	IV	3,104.37 ± 71.10	3,653.95 ± 93.82	−0.08 ± 0.004	0.96	3,149.37 ± 62.26	3,648.05 ± 70.26	−0.07 ± 0.004	0.92
	V	3,964.42 ± 80.73	4,088.84 ± 100.69	−0.01 ± 0.004	0.95	4,029.42 ± 65.11	4,159.79 ± 77.71	−0.02 ± 0.005	0.83
Superior posterior	VI	8,696.00 ± 217.82	8,211.37 ± 173.16	+0.03 ± 0.005	0.93	8,691.11 ± 168.28	8,264.00 ± 154.78	+0.03 ± 0.004	0.91
	Crus I	12,683.68 ± 349.64	13,118.63 ± 297.40	−0.02 ± 0.006	0.92	12,566.79 ± 323.01	12,999.89 ± 292.86	−0.02 ± 0.005	0.92
	Crus II	9,428.84 ± 269.08	9,282.53 ± 235.63	+0.01 ± 0.006	0.93	9,319.11 ± 260.09	9,117.63 ± 229.93	+0.01 ± 0.007	0.86
	VIIb	4,539.05 ± 141.24	4,760.16 ± 162.70	−0.02 ± 0.006	0.94	4,512.95 ± 159.75	4,662.21 ± 143.10	−0.02 ± 0.005	0.96
Inferior posterior	VIIIa	4,848.26 ± 159.25	4,507.37 ± 149.61	+0.04 ± 0.007	0.91	4,797.47 ± 166.02	4,415.21 ± 147.39	+0.04 ± 0.008	0.90
	VIIIb	4,136.21 ± 137.08	4,019.32 ± 124.69	+0.01 ± 0.007	0.90	4,072.89 ± 124.97	4,021.05 ± 127.96	+0.01 ± 0.008	0.88
	IX	3,408.16 ± 124.52	3,613.89 ± 139.02	−0.03 ± 0.006	0.95	3,330.68 ± 99.55	3,589.84 ± 114.48	−0.04 ± 0.005	0.95
	X	710.68 ± 15.81	693.47 ± 16.58	+0.01 ± 0.008	0.75	711.32 ± 15.45	685.68 ± 17.31	+0.02 ± 0.01	0.63
	Total	55,519.68 ± 1,154.79	55,949.53 ± 1,103.89	−0.004 ± 0.002	0.98	55,181.11 ± 921.11	55,563.37 ± 954.48	−0.003 ± 0.003	0.94

Laterality indices (LIs) and correlations (*r*) for left and right cerebellar hemispheres are also reported

To examine the effects of twin handedness on cerebellar volume and asymmetry, ANOVA was conducted with hand (right vs left) and hemisphere (right vs left) as repeated measures. Right and left cerebellar volumes for the combined lobules IV–X were the dependent measures. Lack of significant hand (*F* = 0.60, *df* = 1,18, n.s.), hemisphere (*F* = 3.46, *df* = 1,18, *p* < 0.08) and hand × hemisphere (*F* = 0.02, *df* = 1,18, n.s.) effects indicated that total cerebellar volume did not differ significantly for RH vs LH twins, and differed only marginally as a function of right vs left hemisphere.

ANOVA was conducted with hand (right vs left), hemisphere (right vs left) and region (anterior vs superior-posterior) as repeated measures. Effects were significant for region (*F* = 1442.053, *df* = 1,18, *p* < 0.001), hemisphere (*F* = 41.32, *df* = 1,18, *p* < 0.001) and region × hemisphere (*F* = 29.66, *df* = 1,18, *p* < 0.001). Region effects were due to larger volume of the superior-posterior compared to the anterior lobules (see Table 1). There was an overall pattern of right larger than left hemisphere for the combined volumes; however, the direction and degree of asymmetry varied across regions. The region × hemisphere effects showed a larger right hemisphere for anterior volumes, and near symmetry for superior-posterior ones.

### Cerebellar Asymmetry Correlations with Corpus Callosum Size

**Fig. 2:**
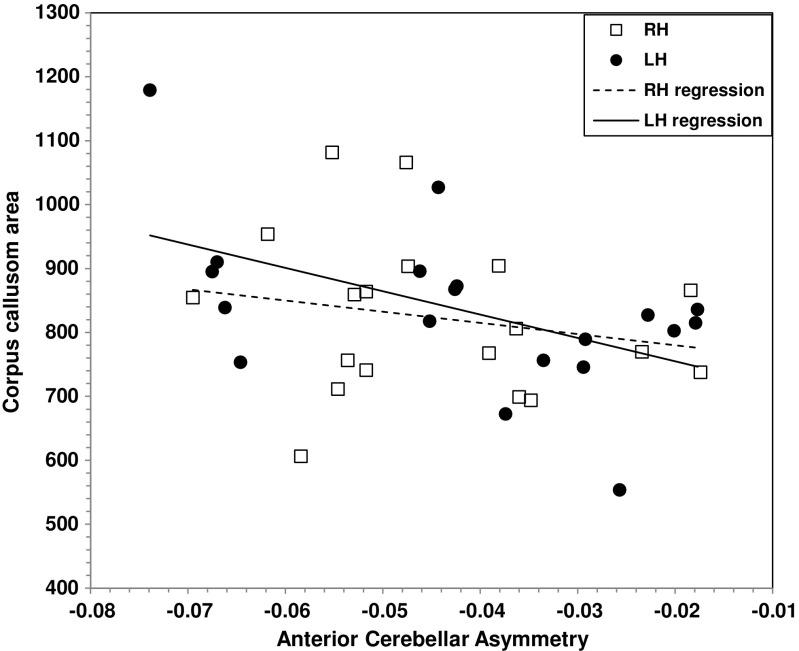
Correlation between corpus callosum area (in mm^2^) and anterior cerebellar asymmetry in LH (filled circles, solid regression line) and RH twins (open squares, dotted regression line) (*LH* left-handed, *RH* right-handed)

Laterality indices were computed for the anterior (IV, V) and superior-posterior (VI, Crus I, Crus II, VIIb) cerebellar volumes, and correlated with CC area for LH and RH twins separately. The correlation between anterior cerebellar asymmetry and CC area was significant in LH (*r = −*0.521, *p* < 0.03; Fig. 2) but not in RH twins (*r = −*0.204, n.s.). CC area did not correlate significantly with superior-posterior cerebellar asymmetry in either handedness group (LH *r = −*0.133; RH *r = −*0.180).

### Anterior Cerebellar Laterality and Regional Corpus Callosum Widths

ANCOVAs were conducted to explore the distinct, handedness-dependent cortico-cerebellar relationship that emerged from the correlations above. Anterior cerebellar volume served as the dependent measure, handedness and hemisphere were the repeated measures and regional CC width factors associated with motor and sensorimotor function served as covariates of interest [53]. The ANCOVA models were based on the prediction that there would be associations between the anterior cerebellum, given its links with cortical motor systems [1, 20], and regions of the CC associated with connectivity between right and left motor cortices [31, 50, 51]. It was predicted that CC widths associated with motor (W22–39, W49–62) and sensorimotor (W65–74) function would covary with anterior cerebellar volume asymmetries to a greater extent than CC W3–18, which reflects prefrontal connectivity, and was not correlated with the other CC regions in this analysis. It was hypothesised that differences between RH and LH twins (handedness factor in the ANCOVA) would emerge in relation to anterior cerebellar asymmetry (hemisphere factor in the ANCOVA), and its relationships with motor and sensorimotor CC regions (covariates W22–39, W49–62, W65–74).

A series of three ANCOVAs was conducted. Anterior cerebellum volume was the dependent measure, with lobule (IV and V), hemisphere (right vs left) and handedness (right vs left) as repeated measures factors, and CC regions as covariates of interest. It is statistically advisable when investigating covariates of interest to minimise inclusion of multiple correlated measures [53]. Thus, covariates were the CC regions which were averaged across RH and LH twins to avoid collinearity, given the high correlations of CC size within twin pairs (correlation coefficients ranged from *r* = 0.626 to *r* = 0.866, *p* values <0.01). In the current model, there were significant correlations (*r* = 0.71 to *r* = 0.86) amongst the three motor and sensorimotor CC regions (W22–39, W49–62, W64–75), so they were examined in separate ANCOVAs. W3–18 however was uncorrelated with these CC regions (*r* = 0.22 to *r* = 0.43). (These CC correlation patterns were reflected in the correlations for LH twins, RH twins and the averaged LH-RH values used as covariates in the ANCOVAs.) CC W3–18 was of particular interest as the region connecting prefrontal cortex, because whilst the anterior cerebellum projects extensively to motor areas there is also evidence for some polysynaptic connection with prefrontal cortex [23]. As such, W3–18 was hypothesised to be neurofunctionally dissociated from the motor and sensorimotor networks between anterior cerebellum and W22–39, W49–62 and W64–75. Therefore, W3–18 was included as a covariate of interest in each of the three ANCOVAs to test this hypothesis. Results are summarised in Table 2.

**Table 2:** Summary of significant ANCOVA results for anterior cerebellar lobules IV and V

	Covariates		
Significant effects	W3–18; W22–39	W3–18; W49–62	W3–18; W65–74
Lobule	*p* < 0.05	*p* < 0.01	*p* < 0.01
Lobule × hemisphere	*p* < 0.01	*p* < 0.01	*p* < 0.01
Handedness × W49–62	–	*p* < 0.05	–
Hemisphere × handedness	*p* < 0.05	*p* < 0.05	*p* < 0.05
Hemisphere × handedness × W22–39	*p* < 0.05	–	–
Hemisphere × handedness × W49–62	–	*p* < 0.01	–
Hemisphere × handedness × W65–74	–	–	*p* < 0.05

Handedness differentiates left- and right-handed twins

Across all ANCOVAs, lobule effects were due to a larger volume for lobule V compared to IV. Lobule × hemisphere effects were due to a larger right-greater-than-left hemisphere difference in lobule IV (mean difference = 524.13; LI = −0.077) compared to lobule V (mean difference = 127.4; LI = −0.016).

An interaction of handedness × W49–62 was present in the analysis which used W3–18 and W49–62 as covariates. Correlations between anterior cerebellar volume and W49–62 were conducted for RH and LH twins, with W3–18 partialled out of the analysis. In LH twins, there was a moderate but non-significant correlation between anterior cerebellar volume and W49–62 (*r* = 0.35). The correlation was near zero for RH twins (*r* = −0.07).

**Fig. 3:**
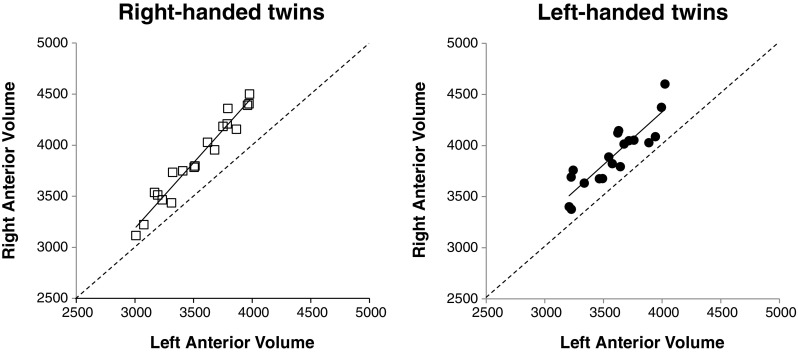
Anterior cerebellar lobule volumes plotted by right and left hemispheres for RH and LH twins. Regressions are plotted as solid lines. The identity lines are plotted as dashed lines. Volumes show averaged measures for lobules IV and V (in mm^3^).

Significant hemisphere × handedness effects were due to a larger right-greater-than-left hemispheric asymmetry in RH twins compared to LH twins for the anterior cerebellum. ANCOVAs comparing right vs left anterior cerebellar volume, whilst covarying for W3–18 with W22–39, W49–62 and W65–74, respectively, showed near significant effects of hemisphere for RH (*F* = 4.10/3.98/4.21, *df* = 1,16, *p* < 0.07) but not for LH twins (*F* = 0.01/0.04/0.04, *df* = 1,16, *p* > 0.85). Means, standard errors and right-left hemisphere correlations for RH twins (right hemisphere = 3871.40 ± 99.96; left hemisphere = 3534.40 ± 73.03; LI = −0.045; *r* = 0.977), compared to LH twins (right hemisphere = 3903.92 ± 70.71; left hemisphere = 3589.40 ± 60.39; LI = −0.042; *r* = 0.884), indicate that the statistical significance of the right-greater-than-left hemisphere difference in the repeated measures comparisons for RH twins may have been enhanced by the higher degree of left-right hemisphere correlation in this group (Fig. 3). Both RH and LH twins’ anterior cerebellar volumes were right lateralised, with all data points falling to the left of the (dashed) identity line. This effect was more marked for RH, particularly at the upper ends of the scales (left hemisphere above 3300 mm^3^; right Hemisphere above 3500 mm^3^).

The higher-order interactions between hemisphere × handedness and the CC covariates (W22–39; W49–62; W65–74) were explored using correlations between the three CC covariates of interest and the anterior cerebellar hemispheres, separately for RH and LH twin groups, with W3–18 partialled out of the analysis (Table 3). In all three analyses, the largest correlation observed was between the callosal motor regions, and the right anterior cerebellar volume of LH twins (W22–39 *r* = 0.386; W49–62 *r* = 0.450; W65–74 *r* = 0.182). The partial correlations of anterior cerebellar volume with CC W49–62 showed the strongest distinction between RH (right hemisphere *r =* −0.062; left hemisphere *r =* −0.078) and LH twins (right hemisphere *r =* 0.450; left hemisphere *r =* 0.208).

**Table 3:** Correlations between CC regions associated with sensori-motor systems (W22–39, W49–62, W65–74) and the anterior cerebellar right and left hemisphere volumes

	RH twins		LH twins	
	Right hemisphere anterior cerebellum	Left hemisphere anterior cerebellum	Right hemisphere anterior cerebellum	Left hemisphere anterior cerebellum
CC W22–39 LH-RH mean	*r* = 0.003	*r* = 0.036	*r* = 0.386	*r* = 0.206
CC W49–62 LH-RH mean	*r* = −0.062	*r* = −0.078	*r* = 0.450 (*p* = 0.061)	*r* = 0.208
CC W65–74 LH-RH mean	*r* = −0.130	*r* = −0.162	*r* = 0.182	*r* = −0.040

CC W3–18, associated with prefrontal cortex, was partialled out. Note that the CC regions consisted of the average of the RH-LH twins’ measurements that were used in the ANCOVAs (see text for details)

*RH* right handed, *LH* left handed

## Discussion

This study revealed a handedness- and region-specific relationship between CC anatomy and the structural laterality of the cerebellum. We used handedness discordant monozygotic female adult twins, and measured MRI indices of CC anatomical structure, as well as cerebellar anatomical volumes. Our main findings showed three differences between RH and LH twins:

1. Anterior cerebellum volume showed a greater degree of right-greater-than-left asymmetry in RH compared to LH twins.
2. The asymmetry of anterior (but not superior posterior) cerebellar volume was correlated with CC area in LH twins only.
3. The asymmetry of anterior cerebellar volume correlated with regional CC widths 49–62 (W49–62) in LH twins only. In LH twins, larger right anterior cerebellum correlated specifically with larger CC region W49–62.

Thus, overall cerebellar volumes were similar in RH and LH twins, with subtle regional differences in asymmetry. RH twins had more asymmetrical anterior cerebellar volumes than LH twins. Although LH twins were less lateralised, their anterior cerebellar asymmetry was associated with CC size, specifically, in regions connecting motor cortex. The primary distinction between RH and LH twins was observed in the degree of coupling between anterior cerebellum hemisphere volumes and the CC. Anterior cerebellar asymmetry was significantly correlated with CC area in LH but not RH twins. Specifically, LH twins with a larger rightward cerebellar asymmetry index had a larger CC area. Further analysis revealed a more regionally specific correlation between the right anterior cerebellar volume and CC region W49–62, connecting left and right motor cortices, but only in LH twins. Given this unique series of genetically near-identical MZHd twins, these findings are consistent with the hypothesis that cortico-cerebellar networks of fine manual motor control may be modulated through environmental influences in a functionally and regionally specific way [22].

There is general consensus emerging that cerebellar computation permits (i) precise timing and automatic temporal sequencing across different cognitive domains [54, 55] including (ii) motor coordination [56], motor control and learning [22]. Debate remains concerning specific cerebellar contributions to particular tasks. Because the microscopic structure and intrinsic neuronal circuitry of the cerebellum are highly preserved across its grey matter, the functional specificity of cerebellar regions occurs by integration into distinct cortico-cerebellar loops in non-human primates [28]. Converging lines of evidence support a similar model of connectivity in humans, which also provides the neuroanatomical basis for cerebellar lateralisation. Resting state fMRI research has revealed networks characterised by direct associations in the degree of cerebellar and contralateral cortical asymmetries [57]. Studies of connections between the cerebellum and cortical areas have independently shown laterality effects in the motor domain [25, 58]. This includes evidence from fMRI and transcranial magnetic stimulation studies of connections between the right cerebellum and the left motor cortex associated with manual movement in right handers [24, 25]. The current study provides further evidence that cerebellar regions are integrated into anatomically distinct functional networks, with anterior lobules participating more directly in lateralised motor control [59]; this is with respect to our specific findings differentiating LH and RH groups by CC motor (but not prefrontal) region correlations with anterior (but not superior-posterior) cerebellar lobes.

A growing body of evidence documents neurostructural and neurofunctional differences between left and right handers [60–63] including cerebellum [57, 64]. Aspects of cognitive task performance, experience, proficiency and age correlated with regional cerebellar volumes in a lateralised manner [65–67]. Our study draws from both literatures to provide a combined examination of (a) lateralisation in the cerebellum; (b) relationships between anatomical asymmetry in the cerebellum and a mediator of cortical asymmetry, the CC; and (c) how these elements and their combinations differ in RH and LH MZHd twins. Anatomical volume differences in anterior cerebellum were subtle, but significantly different, with a greater degree of rightward asymmetry in RH twins. This effect appeared to be driven in part by the higher degree of correlation between right and left anterior cerebellar hemispheres in RH compared to LH twins, rather than purely by a mean difference in overall asymmetry indices. Right-greater-than-left asymmetry in anterior cerebellum was particularly evident in RH individuals with larger volumes (Fig. 3), indicating a possible role for the right hemisphere in determining overall size attainment in this region for RHs. The cerebellum displays particularly rapid growth during late foetal gestation, when the right-greater-than-left asymmetry appears to be established due to more rapid expansion of the right cerebellar hemisphere [68]. Neurogenesis continues in the cerebellum until adulthood. One pathway towards the observed greater degree of right-greater-than-left asymmetry in RH twins may be an experience-dependent maintenance of higher growth rates in the right hemispheric cerebellum, potentially in relation to neural connections with left lateralised cortical systems [57].

Structural imaging studies reflect the presence of experience-dependent changes in the human cerebellum. Its overall volume can increase with intensive practice of motor sequences such as those seen in professional musicians [69]. The implication is that cerebellar regions involved in particular tasks may acquire usage-dependent volume differences. This account is supported by research on the lateralised functional circuitry of the cerebellum, with particular relevance to manual motor learning (see [22] for review). Similar mechanisms of neuroplasticity involving left hand use may account for the differences we observed between RH and LH twins in cortico-cerebellar networks, as reflected in the varying degree of correlation between anterior cerebellar laterality, CC area and W49–62. Wang et al. [57] showed that right handers had stronger cerebro-cerebellar asymmetries compared to left handers, an effect partly reflected in the RH-LH differences in anterior cerebellar asymmetry described above. Moreover, our results seem to indicate that in left handers, the coupling between cerebellar asymmetry and cortical laterality hinges on callosal connectivity in frontal cortex which is distinct in its lateralisation vis-à-vis right handers. There is considerable support for greater incidence of cortical lateralisation profiles in left handers that differ from those in right handers [70], and of accompanying differences in interhemispheric connectivity via the CC. Our study indicates that anterior cerebellar asymmetry plays a role in the organisation of this structure-function network.

The study was designed so that writing hand (+/−2) was used as the participant selection criteria. However, handedness in the broader sense ranged from values of −100 to +100 (RH +70 to +100, LH +20 to −100); therefore, it is not strictly binary as a functional measure of behaviour. Moreover, key relationships reported from the larger study overall pivot on within-group correlations between anatomical and neurofunctional measures. So although groups are defined by the handedness-based distinction within MZHd twin pairs, continuous ranges within these groupings provided the most sensitive outcomes. In the current study, comparisons were designed to contrast the motor (anterior cerebellum and sensori-motor CC regions) to the more cognitively associated (superior posterior cerebellum and prefrontal CC region) with the aim to uncover differences in the underlying cortical-cerebellar relationships related to handedness discordance in MZ twins. To this effect, key differences were discovered in motor systems with distinct patterns of correlation between CC size and cerebellar asymmetry in the LH twins. As part of a larger investigation, the novel comparisons reported here constitute post hoc (rather than a priori) analyses and are constrained to regions and systems of interest relevant to neurocognitive measures reported in relation to handedness comparisons of lateralised cortical language and frontal systems [37, 60, 71].

The functional role of the CC in handedness appears to emerge comparatively late in life [72], i.e. in later childhood when myelination of the CC is approaching adult levels. Cerebellar and cortical white matter mature in a broadly similar age-dependent manner, with a faster maturation during infancy, and slower changes during adolescence/early adulthood [73]. Maturation of cortical circuits follows a hierarchical developmental trajectory—with the primary sensory and motor areas maturing before frontal and temporal association cortices. To some degree, this pattern appears to be under genetic control [74], but with both cortical and cerebellar white matter showing measureable experience-dependent changes [75]. The handedness-specific association between spatially distinct, but functionally correlated components of this network (i.e. anterior cerebellum and motor regions of the CC in LH) is compatible with shared environmental influences shaping their development.

With respect to manual control, our results pertain to one hand (whether it be left or right), rather than of two simultaneously. Unimanual motor function is known to involve lateralised premotor and primary motor cortices, typically linked to cortex contralateral to the hand used [76, 77]. The distinct pattern of covariance that we demonstrated between anterior cerebellum and motor regions of the CC in LH twins is consistent with research showing that variants in the neural basis for lateralised manual motor control [78] and interhemispheric coupling [79] exist in LH individuals. Left hand function is particularly reliant on interhemispheric integration across motor and visuospatial networks. Whilst right side of space is attended to by both cerebral hemispheres, left hemispace is attended to largely by just the right hemisphere. This confers a behavioural advantage to the right hand as it is located naturally in right body-centred hemispace (cf. [80]). The increase in interhemispheric connectivity, as reflected in larger anatomical CC at W22–39 in our LH twin sample [37], could, for example, represent a neuroanatomical substrate for a larger network-specific capacity for hemispheric integration in left handers. Notably, the current study reveals structural relationships in left handers between the CC and the cerebellum which rests on an interhemispheric cortical adaptation indexed to anterior cerebellar asymmetry in that handedness group.

A recent study sampling 2226 singletons (RH *n* = 2307; LH *n* = 119) revealed no significant associations between any volumetric measures of cerebellar lobule asymmetry and handedness, and only a minor degree of correlation between cerebellar and perisylvian cortical volume asymmetry [81]. In contrast, we examined relationships between CC area, regional CC widths and cerebellar volume asymmetries, with a view to understanding links between the cerebellum and interhemispheric cortical connectivity in RH compared to LH MZHd twins. Novel results from our within twin pair study, which is part of a larger project, support and extend earlier work showing distinct structure-function relationships between RH and LH twins. Our evidence provides unique insights because it controls for genetic variability between left and right handers within twin pairs. The subject selection method based on hand preference employed comprehensive questionnaires (augmented by subsequent performance measures), whereby twins were included only if they contrasted maximally on preferred writing hand (i.e. strongly prefer the right (score = +2) or strongly prefer the left (score = −2)). Moreover, our study included detailed anatomical CC measures with the specific aim to investigate interhemispheric cortical connectivity, and a statistical analysis approach to highlight anterior/posterior cerebellar distinctions. Thus, every effort was made to reduce statistical ‘noise’ in the data set via subject selection, design and anatomical focus. Not only did we employ tighter RH-LH subject matching than other studies but also technical matching on MRI acquisition, for which the same 1.5-T MRI scanner was consistently used with identical protocols on the same day, and counter-balanced as to RH-LH twin scanning order. This approach inherently matched the sample for handedness group (with equal numbers of right and left handers), gender and age.

The homogeneity in our sample combined with controls on our methods enhanced the potential for fine-grained detection of effects (i.e. signal) whilst reducing noise due to potential variance swamping. As a result, we were able to detect RH vs LH differences related to cerebellar volume asymmetry and functionally salient regions of the CC. Volume-based differences in cerebellum were subtle—twin handedness affected degree of right-greater-than-left anterior cerebellum and would not necessarily have been detected in a larger group of singletons. However, consistent with our findings, Kavaklioglu et al. [81] found their lowest non-significant *p* value in the comparison of right and left handers in cerebellar lobule V volume, which is in keeping with the location of handedness asymmetry differences in our study’s anterior cerebellar volume. Moreover, Wang et al. [57] reported handedness differences in functional asymmetry, with right handers showing higher rightward asymmetry than left handers in typically rightward cerebellar regions.

In effect, our twin model works with a higher signal-to-noise ratio for handedness against a backdrop of other demographic, biological and experiential factors. Our study’s combination of strong behavioural handedness discordance in a tightly controlled MZ twin study setting of well-matched participants may allow more detailed insights into environmentally driven anatomical correlates of handedness and the role of experience on genetic, plus genetic and environmental intersections, compared to large-scale studies of heterogeneous singleton populations. Indeed, our major finding was not limited to size-based effects of handedness on volume asymmetry; we statistically modelled anatomical connections between anterior cerebellar asymmetry and the CC. The structure-function relationships between handedness and these particular cortico-cerebellar systems have not previously been probed using the approach applied in our study. The results may reflect developmental trajectories and/or the effects of critical periods in development. As indicated above, we do not claim that our results support a particular cause and effect relationship, although we note with some interest that clear anatomical asymmetries in thalamic circuits may also distinguish left-lateralised cerebral asymmetries underpinning language and handedness [82].

As mentioned above, Wang et al. [57] found results consistent in part with one of our main anatomical findings. Specifically, in a comparison of intrinsic functional connectivity in 52 RH and 52 LH singletons, right handers showed stronger functional asymmetry than left handers in cerebellar and cortical regions [37, 57]. This was the case against the larger backdrop of their study showing lower functional asymmetry in cerebellum compared to cerebral cortex and a greater degree of cortico-cerebellar coupling in networks comprised of more strongly lateralised cortical and cerebellar regions. Thus, we also observed stronger cerebellar asymmetry in RH twins, albeit with respect to anterior cerebellar volume (i.e. motor systems). Indeed, we showed significantly greater rightward asymmetry in our anterior compared to superior-posterior cerebellar volume. Hence, the nature and direction of our anatomical effect, but not its location, corresponded with functional asymmetry findings in cortico-cerebellar systems which were more prominent in association compared to motor systems [57]. The novel contribution of our study is its focused anatomical look at the issues of regionally distinct couplings in cortical systems and cerebellar asymmetries from the viewpoint of *interhemispheric cortical connectivity* (via the CC) as a function of handedness. In this context, the association between less lateralised anterior cerebellar volumes and larger CC size fits neatly within the broader understanding of lateralised cortical systems where left handedness is associated with both (i) lower asymmetry and (ii) a greater degree of interhemispheric connectivity. Our work suggests that this extends to cortico-cerebellar networks. Importantly, the right anterior cerebellum shows a greater degree of association with the CC in LH twins. Gheysen et al. [83] showed that the *right* cerebellum plays a key role in the acquisition of manual motor sequencing skills, which suggests a specific role for the right anterior cerebellum in establishing the distinct developmentally based aspects of cortico-cerebellar circuitry observed in LH MZ twins.

Moreover, Wang et al. [57] conclude that “in most people, language processing activates the left inferior frontal gyrus and superior temporal lobe as well as the right cerebellum, including Crus I/II and lobule VI” (p. 46). Results from our current and previous research clearly demonstrate that in MZ handedness discordant females, the left hander does not necessarily match this cortical pattern [37, 60]. Additional work will be needed to better understand the regional, system-based differences in the topography and organisation of functional [57, 84] vs anatomical cerebellar asymmetries [81].

It was beyond the scope of this study to include all possible contrasts between gender and zygosity types. The decision to use only females was opportunistic given their availability in a UK-wide osteoporosis database. Nonetheless, the inclusion of females has several advantages, and the availability of prescreened participants, likewise. Although females tend to have less asymmetric brains than males (cf [85–87], but see [88]), they are more likely to have typical development of language and cortical asymmetry in language regions compared to males [89, 90] and lower incidence of left handedness than males [88, 91]. The UK Twin Registry permitted preselection of participants matched for strength of handedness (i.e. discordant writing hand preference +/−2). Interactions amongst handedness, sex and zygosity will need to be addressed in future studies of cortico-cerebellar networks to explore the generalisability of our findings beyond the demographics described here. Moreover, we caution that this study design does not permit causal inferences underlying the reported correlations. Elmer et al. [92] have also considered the conceptual limitations inherent in using differences in lateralised structure-function relationships of adult groups to infer causal mechanisms based in developmental neuroplasticity (i.e. training/practice).

## Summary

By design, our study does not address the causes of handedness in humans, nor was it designed with the primary aim of mapping motor regions across cortex and cerebellum per se. Rather, it investigates these systems to offer novel insights into environmental (and combined genetic and environmental) contributions to asymmetric cortico-cerebellar motor networks. In the future, these findings may contribute towards new developmental theories on the origins of handedness, which can then be tested specifically on wider populations.

Findings presented here indicate that left handedness can be associated with measurable differences in the anatomical relationships occurring in cortico-cerebellar motor control networks, the anterior ‘motor’ cerebellum and cortical motor fibres of the CC. It is worth noting that handedness based differences in regional cerebellar size were subtle, and partly contingent on factors such as right-left hemisphere correlations. A clearer distinction between RH and LH twins was revealed in relationships between cerebellar laterality and CC size where effects were localised to the right anterior cerebellar hemisphere and CC W49–62 connecting motor cortices. This provides insight into the organisation of cortico-cerebellar motor networks in a tightly controlled cohort of MZHd twins. Our results provide a scaffold for the design of future research on handedness and cortico-cerebellar structure-function relationships from a neurodevelopmental perspective. We advocate contrasting our results to those from non-twin participants in order to investigate cerebellar-callosal correlations and shed further light onto whether the effects of the current report are generalisable to developmental processes in singletons.
